# Rhein Induces a Necrosis-Apoptosis Switch in Pancreatic Acinar Cells

**DOI:** 10.1155/2014/404853

**Published:** 2014-05-13

**Authors:** Xianlin Zhao, Juan Li, Shifeng Zhu, Yiling Liu, Jianlei Zhao, Meihua Wan, Wenfu Tang

**Affiliations:** Pancreatic Diseases Research Group, Department of Integrative Medicine, West China Hospital, Sichuan University, Chengdu 610041, China

## Abstract

*Objectives*. The Chinese herbal medicine Da-Cheng-Qi decoction can regulate a necrosis-apoptosis switch in injured pancreatic acinar cells. This study investigated the effects of rhein, a component of this medicine, on a necrosis-apoptosis switch in pancreatic rat AR42J cells. *Methods*. Cerulein-treated AR42J cells were used. After pretreatment with 479, 119.8, or 29.9 **μ**g/L rhein, cells were cocultured with rhein and cerulein (10^−8^ M) for 4, 8, or 16 h. Apoptosis and necrosis were examined using annexin V and propidium iodide costaining. Mitochondria-dependent apoptosis-associated proteins were examined using enzyme-linked immunosorbent assays and western blotting. *Results*. Few cells died in untreated samples. The number was significantly higher in 16-h-cerulein-treated samples and treatment with 479 **μ**g/L rhein most effectively increased the apoptotic-to-necrotic cell ratio (*P* < 0.05). In cerulein-treated cells, rhein increased the concentrations of p53, cytochrome C, and caspase-3, and increased the Bax/Bcl-2 ratio in a time- and dose-dependent manner, with the maximum effect in cells treated with 479 **μ**g/L rhein for 16 h (*P* < 0.05). *Conclusions*. Rhein induces the necrosis-apoptosis switch in injured pancreatic acinar cells in a time- and dose-dependent manner. Mitochondria-dependent apoptosis signaling pathways might play an important role in this effect.

## 1. Introduction

In severe cases of acute pancreatitis (AP), an inflammatory pancreatic disease, rates of mortality and morbidity are high. The severity of AP is influenced by the balance between two forms of cell death, namely, apoptosis and necrosis. Mild AP is predominantly associated with apoptosis. By contrast, severe AP is predominantly associated with necrosis, which can promote the release of digestive enzymes and inflammatory mediators and ultimately escalate local and systemic inflammatory damage [1, 2]. Therefore, apoptosis induction in injured pancreatic acinar cells might suppress the inflammatory response and alleviate the severity of AP and could thereby be an effective treatment strategy [1–3]. Under certain conditions, a necrosis-apoptosis switch serves to modulate how cells die [4]. In recent years, the role of a necrosis-apoptosis switch and its associated biological mechanism has attracted worldwide attention in the pathophysiology of AP. Consequently, the necrosis-apoptosis switch has been studied in pancreatic acinar cells.

The Chinese herbal medicine Da-Cheng-Qi decoction (DCQD), which was first described in the Shang-Han-Lun in 200 AD, has been widely used throughout China to treat AP for over 30 years [5, 6]. We previously showed that DCQD can ameliorate pancreatic inflammation and pathological damage by inducing a necrosis-apoptosis switch in AR42J cells and a rat model of AP [7]. Other studies similarly reported that treatment with various components of DCQD induces apoptosis and reduces necrosis of acinar cells in AP [8–10]. However, the active components and mechanism underlying this effect remain uncertain. We recently showed that many components of DCQD play a key role in an necrosis-apoptosis switch in vitro and that rhein might be the most bioactive component of this medicine [11]. Rhein can promote apoptosis of cancer cells through the mitochondria-dependent pathway [12, 13]. Mitochondria are the central components involved in apoptosis and changes in mitochondrial morphology are a hallmark of apoptosis [14]. In this study, we investigated the proapoptotic and antinecrotic effects of rhein on AR42J cells and analyzed the molecular mechanism underlying how rhein induces a necrosis-apoptosis switch by examining mitochondria-dependent apoptosis signaling pathways.

## 2. Materials and Methods

### 2.1. Drugs and Reagents

Purified rhein was purchased from the National Institute for the Control of Pharmaceutical and Biological Products (Beijing, China). In our previous pharmacokinetic study, the highest serum concentration of rhein in rats treated with 20 g/Kg/W DCQD was 479 *μ*g/L [15]. Therefore, the purified rhein was reconstituted with fetal bovine serum (PBS) to prepare a 479 *μ*g/L stock solution in dimethylsulfoxide (DMSO) and kept at −20°C. Prior to experiments, the rhein stock solution was diluted in PBS to prepare working solutions (479 *μ*g/L, 119.8 *μ*g/L, and,29.9 *μ*g/L). Dimethylsulfoxide, fetal bovine serum, and Kaighn's modification of Ham's F-12 medium (F12K) were obtained from HyClone (Logan, UT, USA). All antibodies and chemicals were obtained from Sigma (St. Louis, MO, USA). The annexin V-FITC apoptosis detection kit was purchased from Beijing Biosea Biotechnology Co., Ltd (Beijing, China). All enzyme-linked immunosorbent assay (ELISA) kits were purchased from Wuhan ColorfulGene Biological Technology Co., Ltd (Wuhan, China).

### 2.2. Cell Culture and Treatment Groups

Rat pancreatic acinar AR42J cells (CRL-1492, ATCC, Rockville, MD, USA) were seeded at a density of 1 × 10^5^ cells/well in flat-bottom 24-well plates and cultured in F12K supplemented with 10% fetal bovine serum, 100 *μ*g/mL streptomycin, and 100 U/mL penicillin in standard conditions (37°C and 5% CO_2_). AR42J cells (1 million/mL) were divided into the following groups: normal group (NG), in which cells were cultured in medium alone; inflammation group (IG), in which cells were cultured in medium containing cerulein; and treatment groups one (T1), two (T2), and three (T3), in which cells were cultured in medium containing cerulein and 479, 119.8, and 29.9 *μ*g/L rhein, respectively. Experiments were performed 24 h after cells were seeded. To investigate the protective effects of rhein against inflammation, AR42J cells were pretreated with various concentrations of rhein for 30 min and were then coincubated with rhein and cerulein (10^−8^ M) for a further 4, 8, or 16 h. Levels of apoptosis, necrosis, Bax, Bcl-2, p53, cytochrome C, and caspase-3 were examined.

### 2.3. Apoptosis and Necrosis Assays

To determine levels of apoptosis and necrosis, AR42J cells (1 million/mL) were stained using the annexin V-FITC apoptosis detection kit following the manufacturer's instructions. Cells were resuspended in 300 *μ*L binding buffer (10 mM HEPES, pH 7.4, 140 mM NaOH, and 2.5 mM CaCl_2_) containing 5 *μ*L annexin V-FITC and 5 *μ*L propidium iodide (PI) and incubated for 15 min at room temperature in the dark [11, 12]. The suspensions were analyzed by flow cytometry (FAC Scan; Becton Dickinson, USA) to quantitate early apoptotic cells (annexin V-positive and PI-negative) and necrotic or late apoptotic cells (annexin V-positive and PI-positive).

### 2.4. Measurement of p53, Cytochrome C, and Caspase-3 Concentrations by ELISA

To examine concentrations of p53, cytochrome c, and caspase-3 using rat ELISA kits, AR42Jcells (1 million/mL) were prepared following the manufacturer's instructions. Briefly, the sample of cells that was split were added into the specimen diluent. After procedures of incubation, washing, adding enzyme and color, the optical density of each sample at 450 nm was determined using a microplate reader (Bio-Rad, USA) after adding stop solution and within 15 min. The concentrations of p53, cytochrome C, and caspase-3 were calculated using Curve Expert 1.3 software. Take the standard density as the horizontal and the OD value for the vertical and draw the standard curve on graph paper. Find out the corresponding density according to the sample OD value by the sample curve.

### 2.5. Western Blot Analysis of Bax and Bcl-2

Protein levels of Bax and Bcl-2 were examined by western blotting. Briefly, equal amounts of protein (50–80 *μ*g/lane) were separated by sodium dodecyl sulfate-polyacrylamide gel electrophoresis. Proteins were electrophoretically transferred to PVDF membranes (Millipore, MA, USA). Membranes were blocked in Tris-buffered saline containing 0.1% Tween-20 and 5–10% nonfat milk for 1 h and were then incubated with specific rabbit anti-Bax and anti-Bcl-2 primary antibodies [12]. Proteins were visualized by enhanced chemiluminescence (Amersham, Piscataway, NJ) according to the manufacturer's instructions.

### 2.6. Statistical Analysis

Data were analyzed using the Package for Encyclopaedia Medical Statistics (PEMS) 3·1 for windows medical statistics software. Quantitative data are expressed as the mean ± standard deviation (SD.). One-way repeated measures ANOVA followed by multiple pairwise comparisons using the Student-Newman-Keuls procedure were used to analyze differences between treatment and control groups. The level of statistical significance was set at *P* < 0.05.

## 3. Results

### 3.1. Rhein Increases the Ratio of Apoptotic-to-Necrotic AR42J Cells

**Figure 1:**
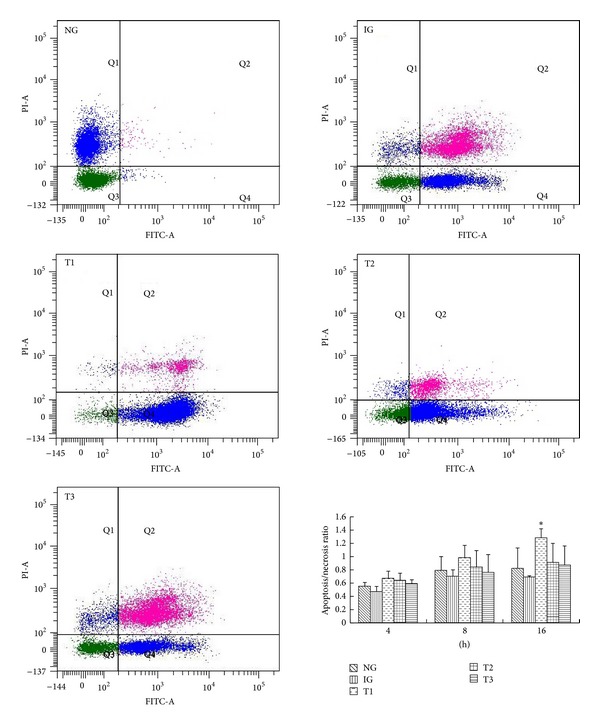
Flow cytometric analysis of the ratio of necrotic-to-apoptotic cerulean-treated AR42J cells cultured with or without various concentrations of rhein. Flow cytometric analysis and histogram of the apoptotic-to-necrotic ratio of AR42J cells in all groups. Results are mean ± SE of three independent experiments. *P < 0.05 compared to IG. NG: normal group, IG: inflammation group, T1: treatment group one, T2: treatment group two, and T3: treatment group three.

The ability of rhein to promote apoptosis in AR42J cells was examined. To this end, annexin V and PI costaining was performed to determine levels of apoptosis and necrosis, respectively. To determine whether rhein treatment has any dose-dependent effects, cells were treated with 479, 119.8, or 29.9 *μ*g/L rhein. These concentrations were chosen based on the maximum serum concentration of rhein in rats determined in our previous pharmacokinetic study [15]. Cells that were annexin V-negative and PI-negative (Q3 region) were regarded as healthy. Cells that were annexin V-positive and PI-negative (Q4 region) were regarded as early apoptotic. Cells that were annexin V-positive and PI-positive (Q2 region) were regarded as necrotic/late apoptotic. In NG, few cells were apoptotic or necrotic. By comparison, there were more necrotic and apoptotic cells in IG (Figure 1). In this group, there were more necrotic cells than apoptotic cells. There were more apoptotic cells and fewer necrotic cells in T1, T2, and T3 than in IG. After 16 h of culture, the ratio of apoptotic-to-necrotic cells was significantly higher in T1 than in IG (*P* < 0.05; Figure 1). At this time point, the ratio of apoptotic-to-necrotic cells was highest in T1, in which cells were treated with the highest concentration of rhein (Figure 1). This indicates that rhein protects against cerulein-induced AR42J cell death in a time- and dose-dependent manner.

### 3.2. Rhein Increases Concentrations of p53, Cytochrome C, and Caspase-3 in Cerulein-Treated AR42J Cells

**Figure 2:**
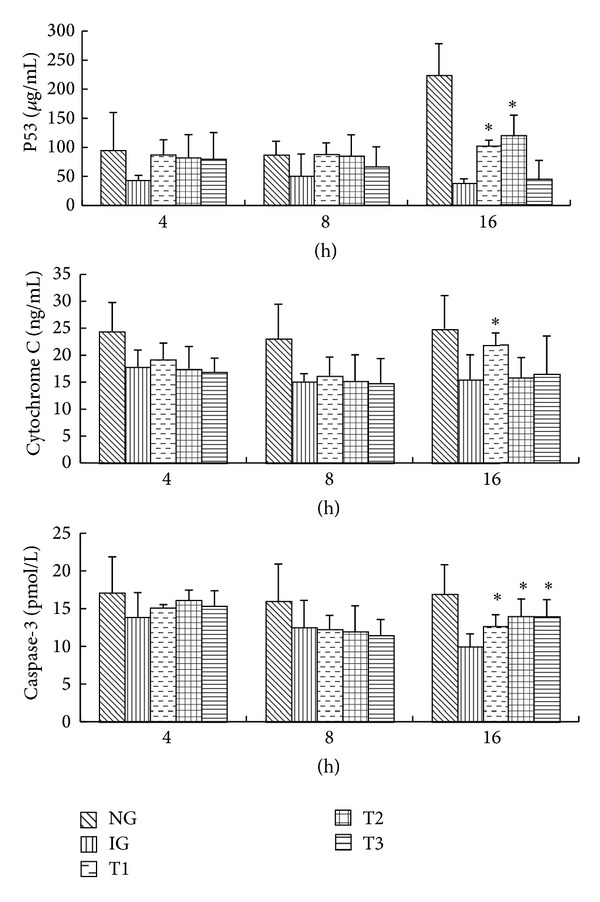
Rhein increases concentrations of p53, cytochrome c, and caspase-3 in cerulein-treated AR42J cells. Histograms of concentrations of p53, cytochrome C, and caspase-3 in all groups. Results are mean ± SE of three independent experiments. *P < 0.05 compared to IG. NG: normal group, IG: inflammation group, T1: treatment group one, T2: treatment group two, and T3: treatment group three.

The molecular mechanism underlying the induction of a necrosis-apoptosis switch by rhein was investigated. To this end, the concentrations of p53, cytochrome c, and caspase-3, which are associated with the mitochondria-dependent apoptosis pathway, were analyzed by ELISAs. The concentrations of p53, cytochrome C, and caspase-3 were lower in IG than in NG. After 16 h of culture, the concentration of p53 was significantly higher in T1 and T2 than in IG (*P* < 0.05; Figure 2; A1 and A2). After 16 h of culture, the concentration of cytochrome c in T1, but not in T2 or T3, was significantly higher than that in IG (*P* < 0.05; Figure 2; B1 and B2). After 16 h of culture, the concentration of caspase-3 was significantly higher in T1, T2, and T3 than in IG (*P* < 0.05, Figure 2, C1 and C2). These results suggest that rhein increases concentrations of p53, cytochrome C, and caspase-3 in cerulein-treated AR42J cells in a time- and dose-dependent manner.

### 3.3. Rhein Increases the Bax/Bcl-2 Ratio in Cerulein-Treated AR42J Cells

**Figure 3:**
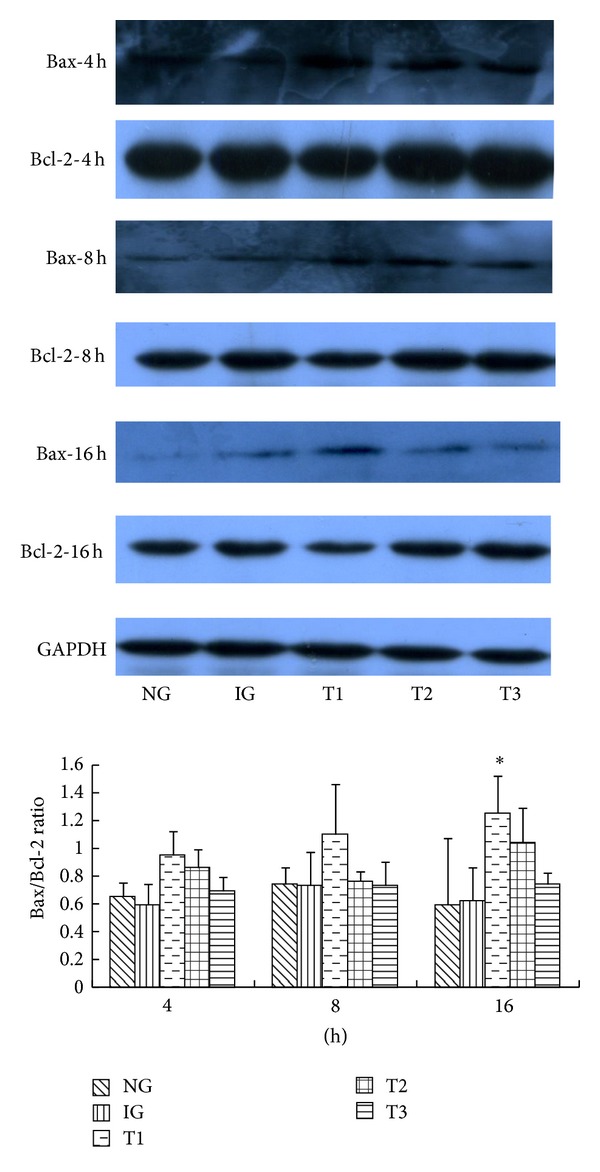
Western blot analysis of Bax and Bcl-2 in cerulein-treated AR42J cells cultured with or without various concentrations of rhein. Western blot analysis of Bax and Bcl-2 in all groups. Histogram of the Bax/Bcl-2 ratio in all groups. Results are mean ± SE of three independent experiments. *P < 0.05 compared to IG. NG: normal group, IG: inflammation group, T1: treatment group one, T2: treatment group two, and T3: treatment group three.

Mitochondrial outer membrane permeabilization (MOMP) is associated with the balance between the proapoptotic protein Bax and the antiapoptotic protein Bcl-2. MOMP can cause the mitochondrial release of cytochrome C, which is a major factor associated with the onset of apoptosis. Therefore, levels of Bax and Bcl-2 were examined by western blotting. Protein levels of Bax and Bcl-2 were higher in IG than that in NG. The level of Bcl-2 was lower and the level of Bax was higher in T1, T2, and T3 than that in IG. The Bax/Bcl-2 ratio was higher in T1, T2, and T3 than that in IG, and this ratio was highest in T1 at the 16 h time point (*P* < 0.05, Figure 3). This indicates that the rhein really enhanced the proportion of Bax/Bcl-2 by increasing the expression of the proapoptotic protein Bax in a time- and dose-dependent manner. This could cause MOMP, resulting in mitochondrial release of cytochrome C and ultimately apoptosis.

## 4. Discussion

This study showed that rhein promotes apoptosis via the mitochondria-dependent pathway and reduces necrosis in cerulein-injured AR42J cells and thereby has a necrosis-apoptosis switch effect. These effects of rhein are similar to those of DCQD; therefore, rhein might be a bioactive component of DCQD and be useful for the treatment of AP.

Our previous studies showed that the traditional Chinese medicine DCQD can regulate necrosis and apoptosis of acinar cells, modulate a necrosis-apoptosis switch to inhibit the inflammatory response, and ameliorate the severity of AP in in vitro and in vivo models [7]. We identified ten components in serum after oral administration of a single dose of DCQD in rats [15]. Some of these components exert anti-inflammatory effects [11], including rhein, magnolol, hesperidin, and naringin, each of which might contribute to regulation of a necrosis-apoptosis switch by DCQD. These findings suggest that rhein is one of the most bioactive components of DCQD [11]. Therefore, we investigated the effects of rhein in an attempt to identify the bioactive components of DCQD that underlie its ability to modulate a necrosis-apoptosis switch and thereby alleviate the severity of AP. Rhein treatment increased the apoptosis index and reduced necrosis in cerulein-treated AR42J cells, and these effects were greatest after culture of 16 h. This showed that rhein induces a necrosis-apoptosis switch in AR42J cells, in agreement with other studies [12, 13, 16]. These findings indicate that rhein contributes to the ability of DCQD to induce apoptosis and regulate a necrosis-apoptosis switch in acinar cells.

To examine the molecular mechanism underlying how rhein regulates a necrosis-apoptosis switch, levels of Bax, Bcl-2, p53, cytochrome C, and caspase-3, which are associated with the mitochondria-dependent apoptosis pathway, were examined. This pathway is dependent on the Bcl-2 family for the mitochondrial release of proapoptotic factors such as cytochrome C [17, 18]. A change in the balance between proapoptotic (e.g., Bax, Bad, and Bid) and antiapoptotic (e.g., Bcl-2 and Bcl-xL) Bcl-2 family proteins can cause MOMP. This allows the mitochondrial release of cytochrome C, which interacts with Apaf-1, to trigger activation of caspases (e.g., caspase-3) and induce apoptosis [14, 19]. The p53 tumor suppressor protein can directly induce apoptosis by modulating the balance between Bax and Bcl-2, which causes MOMP [20, 21]. Rhein treatment significantly increased the concentration of p53 and the Bax/Bcl-2 ratio, which promoted mitochondrial cytochrome C release, caspase-3 activation, and ultimately apoptosis. These effects occurred in a time- and dose-dependent manner and were maximal after treatment for 16 h. In this process of cell apoptosis, Caspase-3 is considered as the most important apoptotic executor and its activation is the symbol of cell apoptosis. Influence of rhein on different proteins in signaling pathway may be different, and the caspase-3 may be the most obvious target protein. Thus, the lower dose rhein can promote the expression of caspase-3, with the increased caspase-3 levels in all three groups (T1–T3) in our study. Another study reported that rhein induces apoptosis by reducing the level of Bcl-2 and thereby altering the Bax/Bcl-2 ratio, leading to a loss of mitochondrial membrane potential, mitochondrial cytochrome C release, and activation of caspase-9 and caspase-3 [13]. These findings demonstrate that rhein induces apoptosis via the mitochondria-dependent pathway. Additionally, reactive oxygen species-mediated activation of NF-*κ*B and p53 signaling pathways might play important roles in rhein-induced apoptosis [12, 16]. The present study focused on the mitochondrial-dependent apoptosis pathway. Further studies are required to investigate other mechanisms by which rhein regulates a necrosis-apoptosis switch in pancreatic acinar cells.

In conclusion, this study suggests that rhein is the primary bioactive component of DCQD responsible for inducing apoptosis via the mitochondria-dependent pathway and thereby plays an important role in modulating a necrosis-apoptosis switch. However, the other bioactive components of DCQD and the molecular mechanisms underlying their effects on a necrosis-apoptosis switch remain poorly understood. Future studies investigating the other bioactive components of DCDQ are required to generate more comprehensive pharmacokinetic and pharmacodynamic data to assist the design and optimization of DCQD formulations.
